# Nanobiomaterials: exploring mechanistic roles in combating microbial infections and cancer

**DOI:** 10.1186/s11671-023-03946-x

**Published:** 2023-12-20

**Authors:** Neha Rawat, Nabeel Ahmad, Pratishtha Raturi, Nirjara Singhvi, Nitin Sahai, Preeti Kothiyal

**Affiliations:** 1 School of Allied Sciences, Dev Bhoomi Uttarakhand University, Dehradun, 248007 India; 2 3D Printing and Visualization Center, University of Pecs, Boszorkany str. 2, Pecs, Hungary; 3 Departmnet of Biomedical Engineering, North Eastern Hill University (Central University), Shillong, India; 4 School of Pharmacy and Research, Dev Bhoomi Uttarakhand University, Dehradun, 248007 India

**Keywords:** Biomaterials, Nano biomaterials, Synthesis, Mechanism of action, Applications, Anticancer, Antimicrobial

## Abstract

The initiation of the "nanotechnology era" within the past decade has been prominently marked by advancements in biomaterials. This intersection has opened up numerous possibilities for enhancing the detection, diagnosis, and treatment of various illnesses by leveraging the synergy between biomaterials and nanotechnology. The term "nano biomaterials" referring to biomaterials featuring constituent or surface feature sizes below 100 nm, presents a realm of extraordinary materials endowed with unique structures and properties. Beyond addressing common biomedical challenges, these nano biomaterials contribute unprecedented insights and principles that enrich our understanding of biology, medicine, and materials science. A critical evaluation of recent technological progress in employing biomaterials in medicine is essential, along with an exploration of potential future trends. Nanotechnology breakthroughs have yielded novel surfaces, materials, and configurations with notable applications in the biomedical domain. The integration of nanotechnology has already begun to enhance traditional biomedical practices across diverse fields such as tissue engineering, intelligent systems, the utilization of nanocomposites in implant design, controlled release systems, biosensors, and more. This mini review encapsulates insights into biomaterials, encompassing their types, synthesis methods, and the roles of organic and inorganic nanoparticles, elucidating their mechanisms of action. Furthermore, the focus is squarely placed on nano biomaterials and their versatile applications, with a particular emphasis on their roles in anticancer and antimicrobial interventions. This review underscores the dynamic landscape of nanotechnology, envisioning a future where nano biomaterials play a pivotal role in advancing medical applications, particularly in combating cancer and microbial infections.

## Introduction

The intersection of nanotechnology and biomedicine has given rise to a burgeoning field of research focused on nanobiomaterials—an innovative class of materials designed to interact with biological systems at the nanoscale. This review delves into the intricate world of Nanobiomaterials, shedding light on their mechanistic roles in combating two critical challenges in healthcare: microbial infections and cancer [1]. In recent years, the relentless emergence of drug-resistant microbial strains and the complexities of cancer demand a paradigm shift in therapeutic approaches. Nanobiomaterials, with their unique physicochemical properties and tailored functionalities, have emerged as promising candidates for addressing these challenges [2]. This review explores the multifaceted applications of nanobiomaterials, unraveling the intricate mechanisms that underpin their efficacy in combating both microbial infections and cancer.

From targeted drug delivery systems that navigate the intricate landscapes of the human body to smart nanosystems that respond dynamically to physiological cues, nanobiomaterials offer a versatile toolkit for precision medicine [3, 4]. Understanding the intricate interplay between these materials and biological entities is pivotal in harnessing their full potential for therapeutic interventions. As we embark on this exploration of nanobiomaterials, we aim to unravel the underlying mechanisms that govern their success in the intricate battlefields of microbial infections and cancer. By bridging the realms of nanotechnology and biomedicine, nanobiomaterials hold the promise of revolutionizing our approach to healthcare, offering hope for more effective, targeted, and less invasive treatments. This review seeks to provide a comprehensive understanding of the current landscape, challenges, and future prospects of nanobiomaterials in the context of combatting microbial infections and cancer.

## Biomaterials

**Fig. 1:**
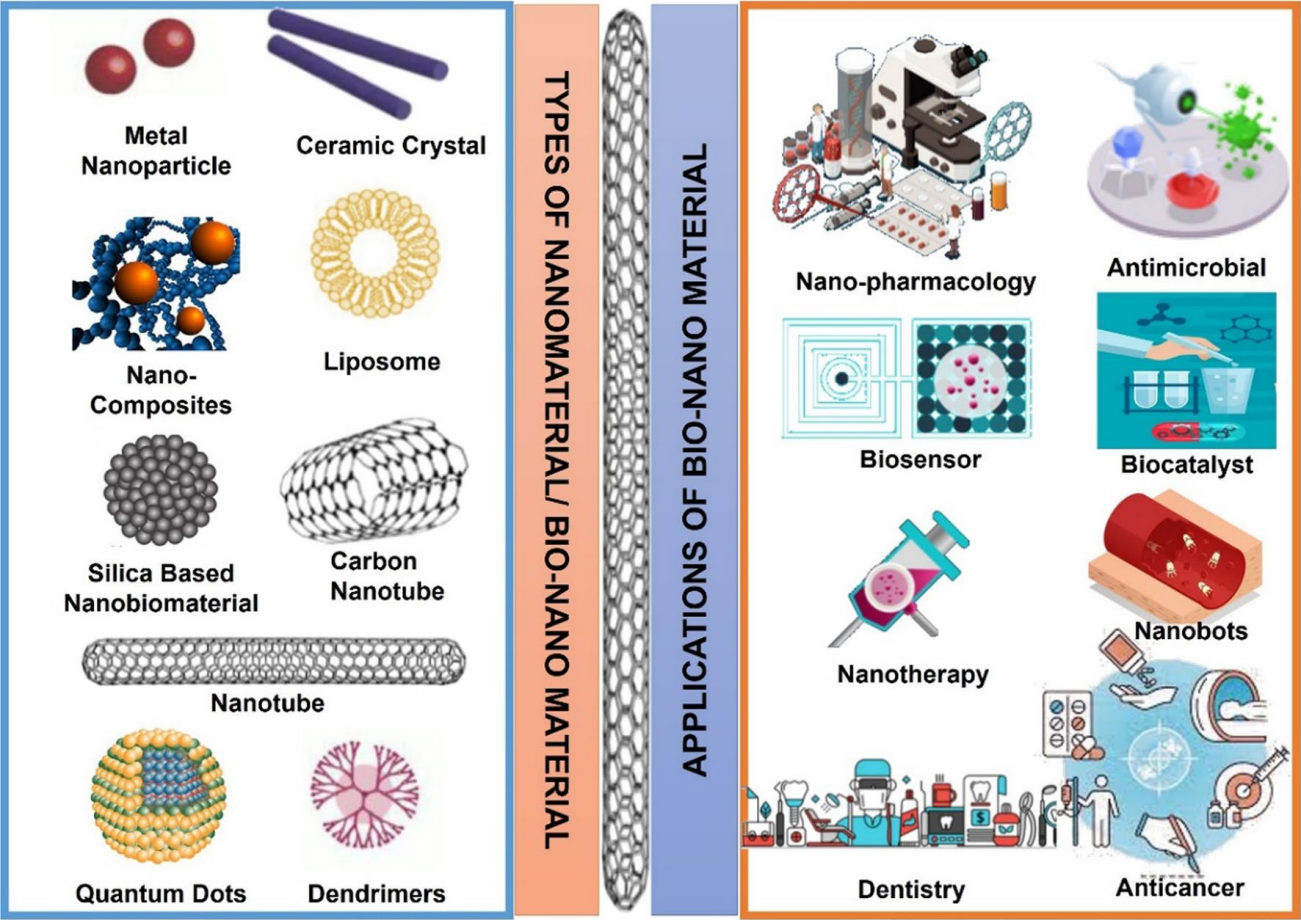
Major types of nanobiomaterial used in the biomedical industry and their applications

A native or synthetic material that can be used for any amount of time to treat, bolster or substitute any organ, tissue, or biological function or to perform a function in close proximity to living tissue is referred to as a biomaterial [5]. In contrast to biological materials produced by a biological system, the term "biomaterial" is typically used to describe materials utilized for biomedical applications [2] (Fig. 1). When seen from the perspective of a material, Nano-biomaterials like nano-membranes, nano-apatite grains and nano-muscle fibres, can be thought of as a composite that makes up biological tissue [3]. The development of nanoparticles, nanofibers, nanocoating, and nanocomposites for use in biomedical applications has been made possible by the rapid expansion of nanotechnology. Due to their special properties, there has been ongoing research on nano-biomaterials for a long time. These applications include bioimaging, medical implants, medication and gene delivery, biosensing, wound healing, tissue engineering, and diagnostic tools such as DNA microarrays and protein [4]. A nano-functionalized surface possesses potential biological capabilities, according to Liu et al.'s research [6]. Producing a surface that is nano-structured can thereby strengthen the clinical applications of biomaterials. Biomaterials are classified into natural, conventional and nano-structured/ nanobiomaterials [7].

### Natural biomaterials

Various subtypes of natural biomaterials are available including protein-based biomaterials (collagen, gelatin, silk), polysaccharide-based biomaterial (cellulose, chitin/chitosan, glucose), tissue/organ-derived biomaterials (decellularized heart valves, blood vessels, etc.). Natural biomaterials are frequently less toxic than synthetic biomaterials, which is one of their main benefits for usage as implants. They are also similar to the host tissues found in the body[6]. In the skin, ligament, bone, tendon, cartilage, and other connective tissues, collagen—mostly often utilized natural polymer—is present. Collagen exists in the body in at least 19 distinct types. These include type I, which is mostly exists in skin, bone & tendons; type II, which is primarily present in joints' articular cartilage; and type III, which is primarily present in blood vessels [6]. Fish collagens have garnered a lot of interest in the past [8] nevertheless, the bulk of commercial collagens are made from pork. Other naturally occurring biomaterials include silicates derived from algae, chitin derived from crustaceans and insects, hair-derived keratin, and plant-derived cellulose, diatoms derived from invertebrates, and calcium phosphates derived from vertebrates [9].

### Conventional biomaterials

Conventional biomaterials have been instrumental in advancing various fields of medicine and engineering, serving as the building blocks for a wide array of medical devices and implants. This review delves into the merits and limitations of these materials, shedding light on their impact on healthcare and biomedical applications. Conventional biomaterials, such as metals (e.g., titanium), ceramics, and polymers, have demonstrated commendable biocompatibility [6]. This property is crucial for minimizing adverse reactions when these materials come into contact with biological systems. Their ability to integrate seamlessly with living tissues has paved the way for successful implants and medical devices. Materials like metals and ceramics offer excellent mechanical strength, making them suitable for load-bearing applications. This characteristic is particularly valuable in orthopedic implants, where durability and stability are paramount for long-term success.

Many conventional biomaterials have been in use for decades, allowing for the accumulation of extensive clinical data and experience. This established track record enhances the confidence of healthcare professionals and researchers in the safety and efficacy of these materials. However, metals, despite their strength, are susceptible to corrosion and wear over time. This can compromise the integrity of implants and may necessitate additional surgical interventions for replacement or repair. Certain biomaterials can trigger inflammatory responses, leading to complications such as fibrosis or rejection [9–11]. Polymers, in particular, may incite immune reactions, challenging their application in some contexts.

### Nanostructured biomaterials/nano biomaterials

**Fig. 2:**
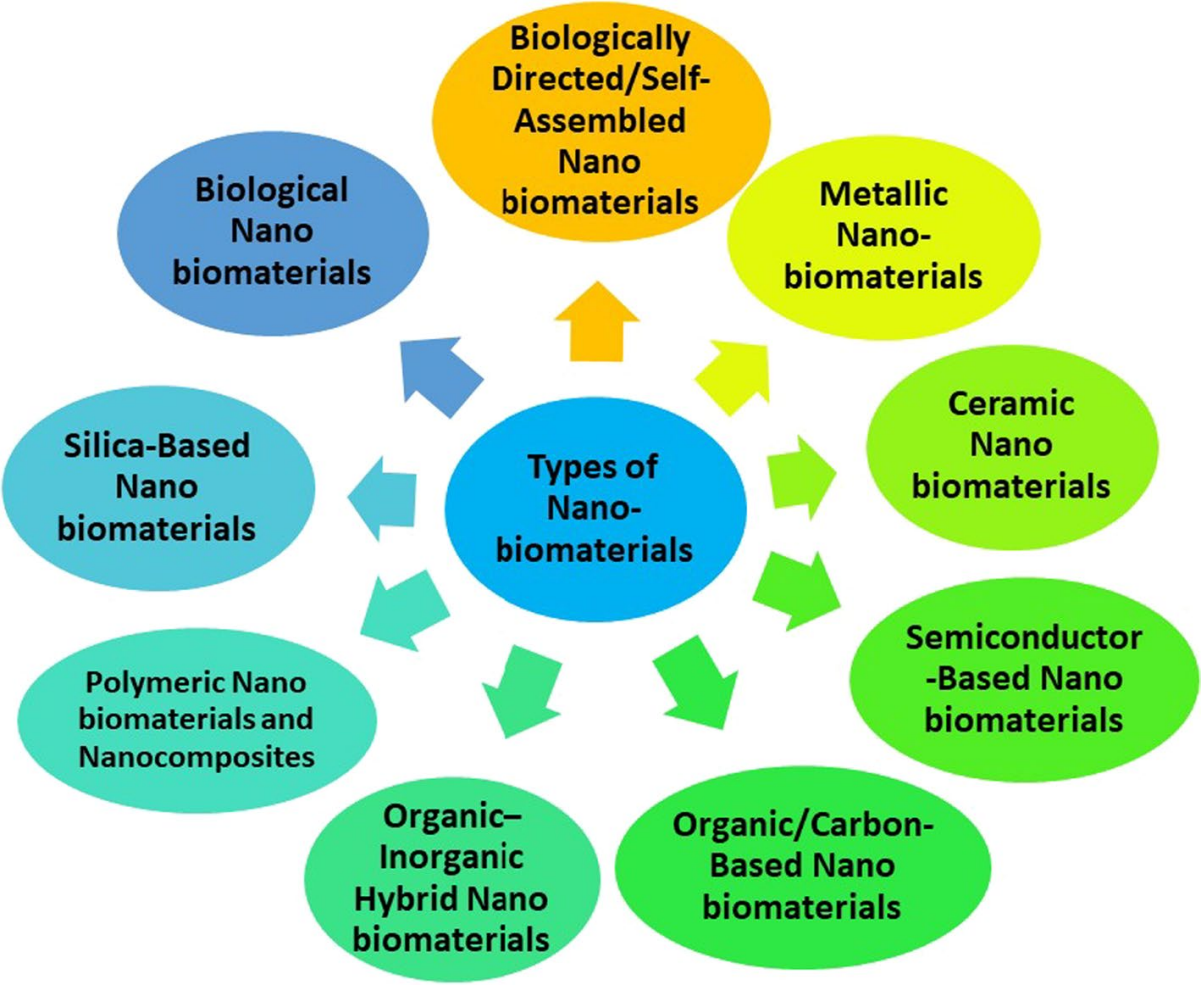
Various types of nano-biomaterials including ceramic, biologically derived, semiconductor, silica based, metallic nanobiomaterials

The word "nano" is a derivative of the Greek word "nano," which means "dwarf." A relatively emerging subject of science is nanotechnology, that is generally speaking, everything that involves developing science and technological advancements between one and one hundred nm with the goal of creating materials, learning fundamental things about their qualities, and using the substances as parts or components to construct brand-new constructions or gadgets. These length scales enable materials to exhibit distinctive features and capabilities [12]. For use in treatment of cancer, medication and delivery of gene, imaging techniques, and other beneficial biomedical applications, biomaterials with a nanostructure, such as nano surfaces, nanoparticles, nanocomposites, and nanofibers like nanoparticles, nanofibers, nano surfaces, and nanocomposites have received a lot of attention. Biomaterials with nanoscale or nanostructure are produced utilizing composite materials, metals, polymers and ceramics. Surface modification methods, such as in situ surface modification, film deposition, and nano coating, can create nano-structured surfaces on conventional biomaterials. The biological functional components have sizes in the nanoscale range, The molecular interactions between nanomaterials and biological systems are therefore not unexpected [13]. Furthermore, nanoparticles possess brand-new electrical, structural, magnetic and optical characteristics that are not present in bulk materials or individual molecules. The study of biomolecules' spatial–temporal interactions at the systemic and cellular levels is one of the main objectives of biology [14]. There are different types of nano-biomaterials such as Ceramic, Biologically derived, Semiconductor, Silica based, metallic nanobiomaterials and others. It would be advantageous to comprehend these interactions since nanomaterials can have molecular interactions with biological systems with tremendous selectivity. A mechanism in order to regulate inherent signals (such as factors that promote growth and signalling molecules) underpinning adult and embryonic stem cells' behaviour could be developed, for example, by comprehending the relationship between stem cells and a specific nano biomaterial [14, 15, 17]. Further, Researchers have been exploring the use of carbon nanofibers (CNFs) in addressing antibiotic resistance. Carbon nanofibers can exhibit antimicrobial properties and have been investigated for their potential use in developing new materials for medical devices, wound dressings, and coatings to prevent bacterial infections [15]. PEEK is a high-performance polymer that has been utilized in the medical field, including oral implants. PEEK's mechanical properties, biocompatibility, and radiolucency make it suitable for dental implants. It has been studied for its use in various dental applications, such as implantable abutments and prosthetic components. Advancements in these areas are likely to continue, and new applications and research findings may have emerged [16] (Fig. 2).

## Synthesis of nanoparticles

Nanomaterials and nanostructures, which operate at the nanoscale dimensions, can be fabricated using various techniques encompassing physical, chemical, and biological methods. These methods enable the transformation of both inorganic and organic materials into nanoscale entities with unique properties and applications. The versatility of nanomaterials is particularly evident in the diverse range of materials that can be engineered at the nanoscale, including inorganic materials such as silicon, metal nanoparticles, and quantum dots, as well as organic materials like micelles, liposomes, polymeric nanoparticles, and dendrimers [18, 19]. Physical methods involve the manipulation of materials at the nanoscale through mechanical or top-down approaches. Techniques such as milling, grinding, and lithography can be employed to reduce bulk materials into nanoparticles. Chemical methods rely on chemical reactions to synthesize nanomaterials, often through bottom-up approaches [20–22]. In the case of inorganic materials, chemical reduction, sol–gel synthesis, and hydrothermal methods are commonly used to create nanoparticles. Biological methods leverage living organisms or biomolecules to create nanomaterials. For example, green synthesis involves using plant extracts or microorganisms to produce nanoparticles, offering an eco-friendly alternative. Nanostructures of various forms, dimensions or chemical compositions can be created with the aim of conjugating with medications of choice, controlled dispersion, both functionalization and target delivery in therapies, depending on the applications and biological consequences. [20, 21].

### Inorganic nanoparticles

Inorganic NPs, particularly MNPs, have provided some extensive applications in drug development because of their distinctive features such as surface plasmon resonance (SPR), among other physio-biochemical characteristics. Superparamagnetic and receptor-mediated targeting effects can be produced using functionalized-iron oxide NPs containing the anti-cancer medication paclitaxel [22]. For improved circulation, biodistribution, superior delivery, and ultimately higher biological effects against cancer, using a polymer to functionalize black phosphorus nanosheet and nano-ceramide-GO NPs are two other potent inorganic nanoparticles [23, 24]. Particularly for the creation of NPs, silver is thought to be the best element, and AgNPs have been seen to exhibit better bactericidal and fungicidal effects because of their greater affinity for microbial cells [19, 25]. As a result, more substances based on silver are being used to reduce the spread of bacteria and inflammation [26–28]. In addition, different silver nanoparticles have been used to cover orthopaedic devices, dental implants, wound dressings, and catheters to prevent microbial infections that are related to them [29]. Silver nanoparticles (AgNPs), while known for their antimicrobial properties, pose potential toxicity concerns for both human health and the environment. Exposure to AgNPs through inhalation, dermal contact, or ingestion may lead to adverse effects on the respiratory and gastrointestinal systems. Additionally, concerns exist regarding genotoxicity, reproductive effects, and the potential for silver accumulation in various tissues, raising questions about long-term exposure consequences for humans. In the environment, AgNPs released into aquatic ecosystems and soils may exhibit toxic effects on aquatic organisms and soil fauna, contributing to ecological imbalances [28].

### Organic nanoparticles

Chitosan nanoparticles, Poly(acrylic acid) (PAA), poly(ethylene glycol) (PEG), Poly-lactic acid (PLA) nanoparticles are examples of biocompatible, biodegradable, and adaptable polymeric NPs that are equally efficient. To reduce their toxicity to cells toward healthy cells and to increase their rehabilitative benefits, NPs are typically either polymer- or peptide-capped [30, 31]. In terms of organic NPs, liposomes have a very distinctive structure. They may also be made in a variety of shapes, sizes, and compositions, and they can include different medicines or bioactive compounds as well as imaging agents or photosensitizers [32]; moreover, they can be devoid of any surface modification or functionalization, or PEG-coated target ligands (peptides, carbohydrates, antibodies and proteins) [30, 33]. They are the perfect carriers for both hydrophilic and hydrophobic medicines (in the aqueous core and lipid bilayer, respectively) [34, 35].

### Mechanistic basis

**Fig. 3:**
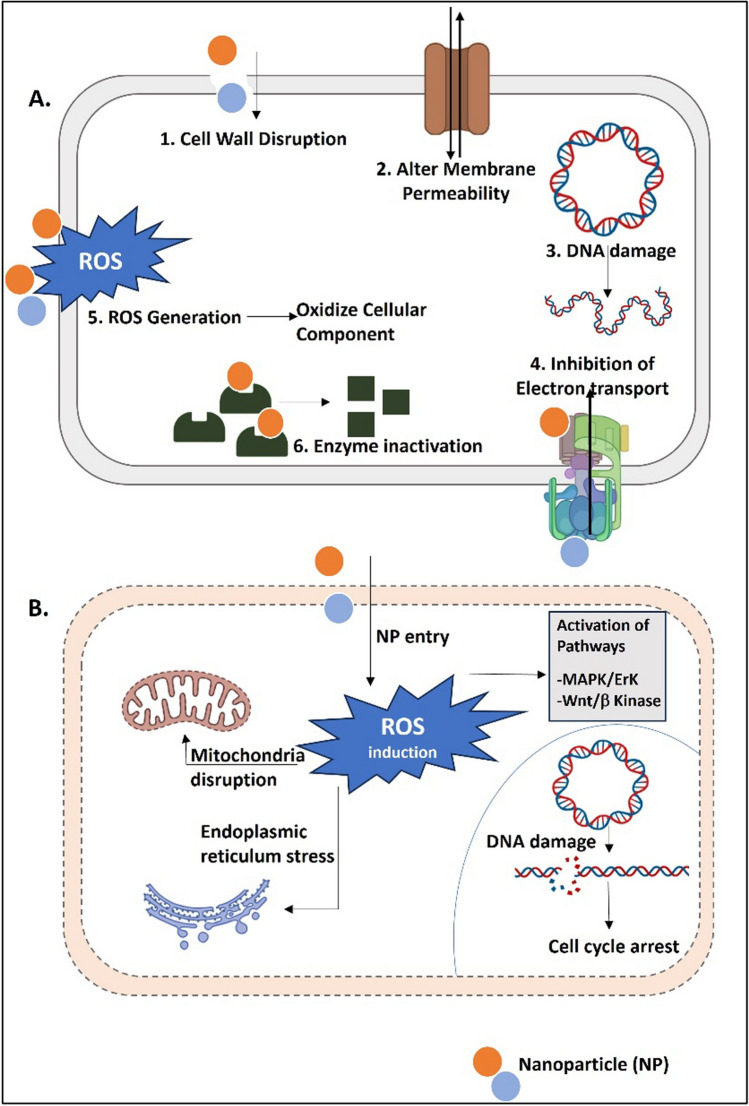
Mechanism of action followed by bionanoparticles as an antibacterial (**A**) and anticancer agent (**B**) [Abbreviations: NP- Nanoparticles, ROS- reactive oxygen species, ErK- extracellular signal-regulated kinase 1/2, MAPK-mitogen-activated protein kinase]

Nucleic acid denaturation, mitochondrial membrane potential disturbance, and damage to lipids, proteins, and mitochondria through the oxidative stress which is caused by the production of reactive oxygen species (ROS) are all possible effects of nanoparticles (NPs) [19, 36] (Fig. 3A); apoptosis, cation accumulation inside of cells, and inflammation have all been shown to be triggered by the production of cytochrome-c [19, 37, 38]. NPs work by impairing cellular integrity [39, 40], inactivating metabolic enzymes of transport chains by engaging with sulfhydryl groups [41], and having an affinity for DNA's phosphorus moiety as well as plasma membrane proteins to inhibit replication [42–44]. AgNPs' biological properties have also been linked to the displacement of Zn^2+^ and Ca^2+^ [45]. As the most effective drug carriers, NPs are capable of delivering medication to the target site or tissue, can provide extended permeability and retention (EPR) effect, and can promote endocytosis [46–49]. NPs can also destroy the biofilm framework and microbial structures [17, 19] (Fig. 3B). Further, the size of nanoparticles plays a pivotal role in determining their fate within the biological milieu. Small-sized nanoparticles often exhibit enhanced permeability and retention (EPR) effects, enabling them to passively accumulate in target tissues, particularly in tumor sites. Conversely, larger nanoparticles may face challenges related to clearance mechanisms, potentially leading to increased accumulation in vital organs. Nanoparticles have demonstrated remarkable success in cancer therapy, with size customization offering precise targeting and improved therapeutic outcomes. For instance, liposomal doxorubicin, with its carefully engineered size, has shown enhanced delivery to tumor tissues, minimizing off-target effects. Similarly, polymeric nanoparticles, such as paclitaxel-loaded micelles, capitalize on size-dependent EPR effects for efficient drug delivery, contributing to improved anticancer efficacy.

## Applications of nano-biomaterials

Amid the surge in digitalization and the digital revolution, characterized by the widespread use of mobile apps, sensors, artificial intelligence (AI), and machine learning for improved record-keeping, data analysis, and risk assessment, a parallel transformation has unfolded in the field of nanotechnology. This evolution is marked by significant advancements in nanomaterials, echoing the transformative impact of digital technologies on various fronts including various applications of nano biomaterials such as antimicrobial application, anticancer application, cosmetics, biocatalyst, drug/ gene delivery, cancer therapy, biosensors, bone/ cartilage regeneration, tissue engineering, anti-infective scaffolds and many more out of which the antimicrobial and anticancer applications of nano-biomaterials are briefed [50]. One notable advancement lies in the realm of targeted drug delivery, where nanomaterials are ingeniously designed to enhance drug efficacy while minimizing side effects. These nanocarriers exhibit remarkable precision in delivering therapeutic agents, promising improved treatment outcomes. Additionally, the convergence of diagnostic and therapeutic functionalities in theranostic nanobiomaterials has garnered attention, enabling real-time monitoring of treatment responses. Tissue engineering has also witnessed significant progress, with nanobiomaterial scaffolds fostering cell growth and tissue regeneration. In the field of cancer nanomedicine, breakthroughs involve the development of nanoparticles that selectively target cancer cells, revolutionizing the precision of cancer therapy. Furthermore, the integration of nanomaterials in biosensors has led to enhanced detection capabilities, impacting areas such as medical diagnostics and environmental monitoring. These breakthroughs collectively underscore the transformative potential of nanobiomaterials in revolutionizing healthcare and medical technologies.

### Anticancer application

More than 10 million fatalities globally (or early one in six deaths) were attributable to cancer in 2020, and this number is expected to increase further, reaching more than 13.1 million in 2030 [51]. The traditional combination of radiation, chemotherapy, or surgery are considered as standard therapies for the treatment of cancers. Cryosurgery has attracted wide attention due to the procedure's several clinical advantages, including the fact that it is less intrusive than typical surgical resection, causes less pain and blood, has a quicker recovery time, costs less, and requires a shorter hospital stay [52]. It is possible to employ argon gas or liquid nitrogen to create extremely cold temperatures during a procedure called cryosurgery. The frozen tissue is either absorbed spontaneously by the body after cryosurgery or it melts and creates a scab [53]. Di and group have reported a novel MgO nanoparticle-based nano-cryosurgical technique for the treatment of cancer. MgO nanoparticles are safe, biodegradable, and have few negative impacts on human health [52]. Both passive and active methods of using nanobiomaterials for cancer treatment are possible. In the passive pathway, nanoparticles move through the bloodstream and aggregate inside of tumors due to increased permeability and retention; in the active pathway, nanoparticles get to the tumor by targeted medication therapy. These nanoparticles can be created using various biomaterials, such as lipids, phospholipids, and polymers, and can have a specified size, electrical charge on the surface, and even surface-applied targeting ligands for site-specific targeting [54]. By changing the medication toxicity profile, nanomaterials offer a significant promise for treating cancer. A better concentration of medications can be delivered to the tumor location while lowering toxicity when using nanoparticles with improved surface properties that can spread more readily within tumour cells. By using nanomaterials with components particular to tumors, cancer cells can be targeted more effectively. Additionally, it avoids the difficulties of indiscriminate anticancer drug biodistribution and large administration dosage. The adoption of anticancer medication delivery via nano methods facilitates the paradigm shift in cancer management [55]. There are numerous broad categories into which nanomaterials employed in cancer therapy can be placed. To combat lack of selectivity and toxicity, bioavailability and increasing the capacity of drug and targeting cancer cells, the microenvironment of tumour and the immune system, these nanomaterials have been altered for a diversity of cancer therapies [56]. It is believed that nanomaterials will transform cancer treatment and diagnosis. The ability of multifunctional nanoscale particles to target tumor sites and then view them using imaging technologies enables the early detection of malignancies. Additionally, for better therapy efficacy, intelligent nano systems can be built as controlled delivery vehicles. These vehicles can bring anticancer medications to a preset spot and then release them at a predetermined rate. These nanomaterials are made of organic or inorganic substances such polymers, carbon nanotubes, quantum dots, superparamagnetic iron oxide, and their composites. They signify fresh approaches to the accurate detection and efficient treatment of cancer [57]. Due to their strong qualities, including autologous pharmaceutical, synergistic effects, biocompatibility, biodegradability, and biosafety, bio-based nanomaterials have drawn a great deal of interest in the field of cancer therapy [58]. Nanoparticles are a superior alternative to microparticles for the treatment of cancer because they are more biodegradable [59]. Due to their thick extracellular matrix, nanoparticles are too large to enter healthy blood vessels [60]. Immature vasculatures produced by tumour-induced angiogenesis restricted lymphatic drainage as the tumour grew [61]. The decreased lymphatic outflow enables nanoparticles to specifically enter cells. The "enhanced permeability and retention effect" (EPR), a phenomenon that has significant implications for passive targeting of nanoparticles, is a key factor [62]. Nanomaterials' superparamagnetic properties make it easier to detect and cure cancer. Superparamagnetic iron oxide nanoparticles, for instance, provide special benefits. They are capable of treating cancer because they are tiny, exhibit strong immune system evasion, and have great targeted specificity [63]. By altering the toxicity profile of the drugs, nanomaterials have a significant potential to impact cancer treatment. The unique physicochemical properties of nanoparticles may introduce novel toxicological effects that are not observed with conventional drug formulations. For example, certain nanoparticles may induce oxidative stress, inflammation, or cellular damage, contributing to toxicity that is distinct from the drug's effects in its traditional form. Further, Machine learning tools and ab initio simulations have increasingly been embraced to enhance data reproducibility, enabling robust quantitative comparisons. These technologies play a pivotal role in facilitating in silico modelling and meta-analyses, thereby making a significant contribution to the advancement of safe-by-design approaches in nanotoxicology and nanomedicine development [64].

The significant advantages of targeted drug delivery via nanocarriers include comparatively high concentration of drug developed at the site of tumour, enhanced colloidal stability and greater drug matter in the formulation. Furthermore, tumour vessels, cell surface receptors and tumour antigens can all be used to accurately and highly affinitive target tumour-specific nanocarriers to cancer cells [65]. Immunotherapy is a two-edged sword that offers the possibility of a full recovery for a small number of patients at the cost of serious and frequently fatal adverse effects [66]. In light of these factors, attempts for utilizing the immune system of the host to produce secure, effective & long-lasting events that are tumoricidal have progressively included tailored systems for improved targeting of particular cells and tissues [67]. Biomaterials and nanoscale science are essential for developing delivery systems which are targeted and offer modular building blocks for developing precise cancer immunotherapies with cellular connections and release patterns that are under control to address a variety of clinical challenges [68]. Nanocarriers can enhance the release of intracellular cargo from Endo lysosomal compartments by a diversity of mechanisms, that includes fusion of membrane or events of disruption, osmotic lysis and formation of pore [69]. Chemotherapy and immunomodulation have both demonstrated to benefit greatly from nanocarriers' ability to allow targeted intracellular drug delivery [70]. These methods can boost the effectiveness of conventional cancer immunotherapies by boosting drug accumulation within the tumour, permitting the codelivery of various medicines and reducing systemic toxicity [71]. The delivery of medications and diagnostics to certain biological targets can be stabilized using nano biomaterials, either singly [72] or in combination [73]. It is extremely difficult to load and encapsulate these payloads at the ideal concentrations inside nanocarriers due to their various physicochemical properties, which include proteins, small molecule medicines, and nucleic acids. Using techniques like nanoprecipitation has made it simpler to load many payloads into nanocarriers [74, 75] and microfluidics [76]. The transporting and delivery of anticancer medications (Doxorubicin and Sorafenib) have been observed to be improved when PEG coupled with beta-Cyclodextrin [77, 78]. The PEG-PCL (PEG coupled with beta-Caprolactone) copolymer has been specifically designed to deliver hydrophobic medications or biomolecules (such as cytokines) against many types of malignancies [79–82]. Additionally, it has been observed that the amphiphilic block copolymer composed of PEG as the shell and PAA as the core enhances the distribution of the anticancer medication doxorubicin with EPR effects [83, 84]. Currently, the use of nanoparticles with Docetaxel and other molecules aims to overcome the resistance developed by cancer cells. Docetaxel has been enclosed in hydrophobic NPs made of PEA for biological effects against lung malignancies. Glycopolymer-functionalized NPs were created to increase the triggered release of biomolecules or medications to cancer locations [85]. The primary goal of anticancer medication functionalization is focused delivery to the sick or desired spot while also reducing leakage to neighbouring tissues [86, 87]. Rapamune (micelles with rapamycin) and Abraxane (albumin with NPs), two other nanobiotechnology-based anticancer medications, have increased anticancer effects and lowered cytotoxicity to normal cells [88]. Cancer cells' intracellular space can be diffused with NPs to produce an EPR effect [89]; When used to trigger apoptosis in cancer cells, biogenic MNPs are particularly successful. It is clear that biogenic AgNPs can activate the caspase-3 intrinsic pathway to trigger apoptosis in malignant cells more effectively than healthy ones. As opposed to immunomodulation, which is used to stimulate or suppress the immune system using natural or manmade bioactive chemicals or medications to treat infections or cancer. Immunotherapy works to stimulate the body's defense mechanisms, either innate or adaptive, so they can identify and destroy malignant or tumor cells [90–96].

### Antimicrobial application

A novel class of antimicrobial drugs known as nanomaterials has different mechanisms of action from traditional antibiotics. Engineered nanoparticles have potential utilization in a multiple of consumer goods that includes packaging, water treatment and medicinal. Microorganism antibiotic resistance is irrelevant for manufactured nanoparticles [97]. Due to their size and capacity to damage cells through a variety of methods, nanoparticles have been demonstrated to own anti-bacterial effectiveness toward a diversity of disease types. Nanomaterials offer an intriguing chance to restrict microbial development before human infection, in contrast to antibiotics, which are given to patients to treat illnesses and infections. Due to this, several applications have been targeted by the development of engineered antimicrobial materials, where the active antimicrobial agents are nanoparticles [98]. The creation of novel, "out of the box," therapies is necessary in order to treat bacterial infections that are contrary to antibiotics due to formation of biofilm or acquired resistance. The use of therapeutics based on nanomaterials has promise for treating infections caused by bacteria that are challenging to treat because they can work around established defences against drug resistance. Additionally, nanoparticles have the capacity to target biofilms and defeat refractory diseases due to their distinct size and physical characteristics [99]. Alternative antibacterial therapies have been developed as a result of the growth in bacteria which are antibiotic-resistant, particularly resistant strains to last-resort treatments & the bounded efficacy of antibiotics in eradicating biofilms. Antibacterial biomaterials such as polycationic polymers and biomaterial-aided delivery of non-antibiotic therapies such as antimicrobial enzymes and peptides, bacteriophages, have made it easier to treat infections that are resistant to antibiotics and that recur. Biomaterials provide prolonged release at the infection site in addition to tailored administration of various medicines, minimizing any potential systemic side effects [100]. Metal-based NP have been thoroughly studied for a number of biomedical applications. Additionally, to their decreased size and bacterial selectivity, metal-based NP, according to the World Health Organization, have shown effectiveness against pathogens that have been designated as priorities [101]. Due to their inability to bind to a particular bacterial cell receptor, it is well recognized that metal-based nanoparticles can be hazardous to bacteria in general. As a result, it is harder for germs to develop resistance to them, and their antibacterial action is more comprehensive. So far, the great majority of studies on the effectiveness of metal-based nanoparticles have demonstrated great results in both Gram-negative and Gram-positive bacteria [101]. To counteract the resistance developed by numerous pathogenic microorganisms against the majority of commonly prescribed antibiotics, there has been a notable rise in interest surrounding unconventional antibiotic compounds. This increased attention stems from the need to explore alternative therapeutic strategies that can effectively target and combat antibiotic-resistant strains of bacteria, viruses, and other pathogens. Particularly, numerous antimicrobial nanoparticles and carriers for administering antibiotics at the nanoscale have shown promise in curing infectious illnesses, such as those that are resistant to antibiotics, in both animal models and in vitro [102]. Acknowledging to endogenous or outer stimuli (i.e., low pH, enzymes or light), nano systems can efficiently concentrate at the infected area, provide bactericidal activity that is multifaceted and synergistic and permit controlled antibiotic release. Particularly, the nano-platform which is coupled with photothermal therapy/photodynamic therapy can improve the killing of bacteria and entrance or removal of biofilm. Additionally, nano-particle-based strategies that involved anti-virulence, bacterial killing, and other mechanisms were also utilized [103]. Due to their small size, the nanoparticles are perfect for biological processes used in the fight against microorganisms. Metals including zinc, iron, silver & copper nanoparticle kinds have shown a great deal of potential as bactericidal and fungal agents, indicating their potential for use as efficient antibacterial reagents in the treatment of wounds and related medical conditions [101]. Against various pathogenic bacterial and viral species, these nanomaterials demonstrated antibacterial action. In contrast to small molecule antimicrobial treatments, which exhibit transient antimicrobial activity and environmental toxicity, nanomaterials offer extended antimicrobial action with little toxicity. They are therefore a potential foundation for alternative bacterial infection management techniques [101]. The antimicrobial nanoparticle physically harms the organism's cell membrane in order to prevent the development of drug-resistant germs [104]. By enhancing the therapeutic efficacy of existing antimicrobial medications, biocompatible nanomaterials offer possible methods for reducing drug resistance in bacteria. Nano vehicles (NVs), carriers based on nanotechnology, offer physicochemical properties which are unique like very small and adjustable size, dominant reactivity, an elevated surface area to volume ratio and functionalized form. The aforesaid disadvantages of conventional antimicrobial therapy can be overcome by the antimicrobial nano vehicles, which can facilitate and particularly target the antimicrobial medicines [105]. One of the most efficient NPs-based drug delivery methods for prolonged release of traditional antibiotics without raising concentration is liposomes. Multiple NPs have been investigated and tested against microbial infection, including MDR bacterial strains[106]. Liposomes are a great nanocarrier for anti-fungal medications like amphotericin B because of their structure and properties, which lower their cytotoxicity; It has been discovered that organic NPs such chitosan nanoparticles are efficient against MDR infections like Neisseria gonorrhoeae [107]. AgNPs have been employed in the creation of implant materials by fusing them with anti-inflammatory [110], anti-fungal [109], polymers [108], antibacterial [17, 19], and antiviral drugs [111]. Additionally, chemically produced nanocomposites containing silver, fluoride, and chitosan have demonstrated potent antibacterial properties against pathogenic *Candida* and *Enterococcus* species [112]. Some nano-biomaterials along with its applications are shown in the form of a table (Table 1).

**Table 1:** Representation of few nanobiomaterials along with its applications

Nanomaterial	Application	References
Nanotubes	Biosensors	[133]
	Neuron scaffolds	
Nanospheres	Monitoring cancer relapse though cell targeting	[129–132]
	Wound healing application	
Nano silica	Drug delivery	[125–127]
	Bioimaging	
Silver nanoparticle	Antimicrobial activity against *E. coli and others*	[47, 115–124]
	Tissue repair and regeneration	
Nanofibers	Drug delivery	[128]
	Tissue repair	
Nanocomposite	CNT presents antimicrobial activity against *S.aureus and others*	[113, 114]
Fullerenes	Pharmaceutical	[133]
Carbon coated nanobelts and nanoplates	Electronic structure	[133]
	Semiconductor membrane	

## Challenges in combating microbial infections and cancer

The challenges and limitations associated with the use of nanobiomaterials in combating microbial infections and cancer are multifaceted. One key concern is the potential toxicity of nanoparticles, which can vary based on their composition and size. Achieving optimal biocompatibility while maintaining therapeutic efficacy is a complex balance that necessitates a deeper understanding of the interactions between nanobiomaterials and biological systems [113]. Precise targeting of infected or cancerous cells is another challenge, as the biodistribution of nanoparticles within the body may not always align with therapeutic objectives, leading to off-target effects. Additionally, the scalability and cost-effectiveness of nanobiomaterial production present practical challenges that need to be addressed for these technologies to be widely accessible [114]. The dynamic nature of biological systems introduces further complexities. Designing nanobiomaterials capable of effectively navigating physiological barriers and responding to the intricate signaling pathways involved in infection and cancer requires a nuanced approach. Standardized testing methods and regulatory frameworks are essential to ensure the safety and efficacy of nanobiomaterials in clinical applications [47].

## Conclusion

Nano biomaterials refer to particles and devices crafted within the nano-size range of 1–100 nm, specifically designed for biomedical or biological purposes. These materials are categorized primarily based on their composition, falling into groups such as silica-based, metallic, carbon-based, semiconductor-based, and polymeric nano biomaterials. Alternatively, their structural characteristics allow them to be classified as tube structures or other sophisticated nano biomaterials. The ongoing exploration of nano biomaterials is driven by their distinctive features, and their applications span various domains, including bioimaging, medical implants, drug and gene delivery, biosensing, wound healing, tissue engineering, and diagnostic tools like DNA microarrays and proteins. The continuous study of nano biomaterials holds promise for revolutionary advancements in the medical industry, offering a platform for the development of superior treatments. A specific area of urgent attention is the construction of nano-antimicrobial biomaterials. This mini review provided a succinct overview of biomaterials and their types, synthesis processes, organic and inorganic nanoparticles, elucidating their mechanisms of action. The focal point is on nano biomaterials and their diverse applications, with particular emphasis on their roles in anticancer and antimicrobial interventions. This review anticipated that the evolving landscape of nanotechnology will pave the way for innovative and impactful contributions to medical treatments, particularly in the realms of cancer therapy and combating microbial infections.

## Data Availability

Rawa data is available upon request from the corresponding author.

## Code availability

Source code is available upon request from the corresponding author.

## References

[1] Zhang X, Wang X, Jiao W, Liu Y, Yu J, Ding B (2023). Evolution from microfibers to nanofibers toward next-generation ceramic matrix composites: a review. J Eur Ceram Soc.

[2] Wang M, Shi D (2004). Bioactive materials and processing. Biomaterials and tissue engineering.

[3] Gupta KK, Dhoble SJ, Krupski AR (2020). Facile synthesis and thermoluminescence properties of nano bio-ceramic β-Ca_2_P_2_O_7_:Dy phosphor irradiated with 75 meV C^6+^ ion beam. Sci Rep.

[4] Patil-Sen Y (2021). Advances in nano-biomaterials and their applications in biomedicine. Emerg Top Life Sci.

[5] Ahmad N, Bhatnagar S, Saxena R, Iqbal D, Ghosh AK, Dutta R (2017). Biosynthesis and characterization of gold nanoparticles: kinetics, in vitro and in vivo study. Mater Sci Eng C.

[6] Festas AJ, Ramos A, Davim JP (2020). Medical devices biomaterials—a review. Proc Inst Mech Eng Part L J Mater Des Appl.

[7] Roy AK, Jones III AA, Webster TJ. Translational medicine and biomaterials: basics and relationship. In: Biomaterials in translational medicine. Academic Press; 2019. pp. 1–22. https://www.sciencedirect.com/science/article/abs/pii/B9780128134771000013?via%3Dihub.

[8] Almeida AP, Saraiva JN, Cavaco G, Portela RP, Leal CR, Sobral RG, Almeida PL (2022). Crosslinked bacterial cellulose hydrogels for biomedical applications. Eur Polym J.

[9] Troy E, Tilbury MA, Power AM, Wall JG (2021). Nature-based biomaterials and their application in biomedicine. Polymers.

[10] Stewart MG, Bagby M (2020). Clinical aspects of dental materials.

[11] Punj S, Singh J, Singh K (2021). Ceramic biomaterials: Properties, state of the art and future prospectives. Ceram Int.

[12] Avi PK, Patel SC, Sitharaman B. Nano biomaterials: current status and future prospects. Nano biomaterials handbook Edited by Balaji Sitharaman. CRC Publication; 2011.

[13] Kumar V, Choudhary AK, Kumar P, Sharma S (2019). Nanotechnology: nanomedicine, nanotoxicity and future challenges. Nanosci Nanotechnol Asia.

[14] Singh A, Amiji MM (2022). Application of nanotechnology in medical diagnosis and imaging. Curr Opin Biotechnol.

[15] Smith JA (2020). Antibiotic resistance mitigation using carbon nanofibers. J Nanomed.

[16] Baig N, Kammakakam I, Falath W (2021). Nanomaterials: A review of synthesis methods, properties, recent progress, and challenges. Mater Adv.

[17] Dwivedi M, Singh SL, Bharadwaj AS, Kishore V, Singh AV (2022). Self-assembly of DNA-grafted colloids: a review of challenges. Micromachines.

[18] Dutt Y, Pandey RP, Dutt M, Gupta A, Vibhuti A, Raj VS, Chang CM, Priyadarshini A (2023). Silver nanoparticles phytofabricated through *Azadirachta indica*: anticancer, apoptotic, and wound-healing properties. Antibiotics.

[19] Dutt Y, Dhiman R, Singh T, Vibhuti A, Gupta A, Pandey RP, Raj VS, Chang CM, Priyadarshini A (2022). The association between biofilm formation and antimicrobial resistance with possible ingenious bio-remedial approaches. Antibiotics.

[20] Sanità G, Carrese B, Lamberti A (2020). Nanoparticle surface functionalization: how to improve biocompatibility and cellular internalization. Front Mol Biosci.

[21] Thakur V, Kutty RV (2019). Recent advances in nanotheranostics for triple negative breast cancer treatment. J Exp Clin Cancer Res.

[22] Wang B, Wu W, Lu H, Wang Z, Xin H (2019). Enhanced anti-tumor of pep-1 modified superparamagnetic iron oxide/PTX loaded polymer nanoparticles. Front Pharmacol.

[23] Gao N, Nie J, Wang H, Xing C, Mei L, Xiong W, Zeng X, Peng Z (2018). A versatile platform based on black phosphorus nanosheets with enhanced stability for cancer synergistic therapy. J Biomed Nanotechnol.

[24] Wang SB, Ma YY, Chen XY, Zhao YY, Mou XZ (2019). Ceramide-graphene oxide nanoparticles enhance cytotoxicity and decrease HCC xenograft development: a novel approach for targeted cancer therapy. Front Pharmacol.

[25] Khatoon A, Khan F, Ahmad N, Shaikh S, Rizvi SMD, Shakil S, Al-Qahtani MH, Abuzenadah AM, Tabrez S, Ahmed AB, Alafnan A, Dutta R (2018). Silver nanoparticles from leaf extract of *Mentha piperita*: eco-friendly synthesis and effect on acetylcholinesterase activity. Life Sci.

[26] Gholami A, Rasoul-amini S, Ebrahiminezhad A, Seradj SH, Ghasemi Y (2015). Lipoamino acid coated superparamagnetic iron oxide nanoparticles concentration and time dependently enhanced growth of human hepatocarcinoma cell line (Hep-G2). J Nanomater.

[27] Choi Y, Ryu GH, Min SH, Lee BR, Song MH, Lee Z, Kim BS (2014). Interface-controlled synthesis of heterodimeric silver–carbon nanoparticles derived from polysaccharides. ACS Nano.

[28] Rizzello L, Pompa PP (2014). Nanosilver-based antibacterial drugs and devices: mechanisms, methodological drawbacks, and guidelines. Chem Soc Rev.

[29] Ahmad N, Mohd S, Rizvi D, Sahai N, Dutta R (2016). Biosynthesis, characterization of gold nanoparticles using *M. indica* leaf extract and their anticancer activity. Int J Nanomed.

[30] Kumar A, Sharipov M, Turaev A, Azizov S, Azizov I, Makhado E, Rahdar A, Kumar D, Pandey S (2022). Polymer-based hybrid nanoarchitectures for cancer therapy applications. Polymers.

[31] Xu M, Liu J, Xu X, Liu S, Peterka F, Ren Y, Zhu X (2018). Synthesis and comparative biological properties of Ag-PEG nanoparticles with tunable morphologies from janus to multi-core shell structure. Materials.

[32] Yetisgin AA, Cetinel S, Zuvin M, Kosar A, Kutlu O (2020). Therapeutic nanoparticles and their targeted delivery applications. Molecules.

[33] Guimarães D, Cavaco-Paulo A, Nogueira E (2021). Design of liposomes as drug delivery system for therapeutic applications. Int J Pharm.

[34] Szoka FC. Liposomal drug delivery: current status and future prospects. In: Membrane fusion; 2019. pp. 845–90. https://www.taylorfrancis.com/chapters/edit/10.1201/9780367811525-36/liposomal-drug-delivery-francis-szoka.

[35] Samimi S, Maghsoudnia N, Eftekhari RB, Dorkoosh F. Lipid-based nanoparticles for drug delivery systems. In: Characterization and biology of nanomaterials for drug delivery; 2019. pp 47–76. https://www.semanticscholar.org/paper/Lipid-Based-Nanoparticles-for-Drug-Delivery-Systems-Samimi-Maghsoudnia/0914378f43062762466b6b9bd7b74a9a2b04a11e.

[36] Nayak D, Kumari M, Rajachandar S, Ashe S, Thathapudi NC, Nayak B (2016). Biofilm impeding AgNPs target skin carcinoma by inducing mitochondrial membrane depolarization mediated through ROS production. ACS Appl Mater Interfaces.

[37] Rizvi SMD, Hussain T, Alshammari F, Sonbol H, Ahmad N, Faiyaz SSM, Kamal MA, Khafagy ES, Moin A, Abu Lila AS (2023). Nano-conversion of ineffective cephalosporins into potent one against resistant clinical uro-pathogens via gold nanoparticles. Nanomaterials (Basel, Switzerland).

[38] Bi S, Ahmad N (2022). Green synthesis of palladium nanoparticles and their biomedical applications. Mater Today Proc.

[39] Parthasarathy A, Vijayakumar S, Malaikozhundan B, Thangaraj MP, Ekambaram P, Murugan T, Velusamy P, Anbu P, Vaseeharan B (2020). Chitosan-coated silver nanoparticles promoted antibacterial, antibiofilm, wound-healing of murine macrophages and antiproliferation of human breast cancer MCF 7 cells. Polym Test.

[40] Ahmad SA, Das SS, Khatoon A, Ansari MT, Afzal M, Hasnain MS, Nayak AK (2020). Bactericidal activity of silver nanoparticles: a mechanistic review. Mater Sci Energy Technol.

[41] Shaheen U, Samad A, Manzoor F, Sheikh IS (2021). Biocidal activity of silver nanoparticles against *Escherichia coli*. Pak Euro J Med Life Sci.

[42] Kumar A, Devi M, Kumar M, Shrivastava A, Sharma R, Dixit T, Singh V, Shehzad K, Xu Y, Singh K, Hu H (2022). Silicon nanostructures and nanocomposites for antibacterial and theranostic applications. Sensors Actuators A Phys..

[43] Hoseinnejad M, Jafari SM, Katouzian I (2018). Inorganic and metal nanoparticles and their antimicrobial activity in food packaging applications. Crit Rev Microbiol.

[44] Gouyau J, Duval RE, Boudier A, Lamouroux E (2021). Investigation of nanoparticle metallic core antibacterial activity: gold and silver nanoparticles against Escherichia coli and Staphylococcus aureus. Int J Mol Sci.

[45] Breisch M, Grasmik V, Loza K, Pappert K, Rostek A, Ziegler N, Ludwig A, Heggen M, Epple M, Tiller JC, Schildhauer TA, Sengstock C (2019). Bimetallic silver–platinum nanoparticles with combined osteo-promotive and antimicrobial activity. Nanotechnology.

[46] Jandt KD, Watts DC (2020). Nanotechnology in dentistry: present and future perspectives on dental nanomaterials. Dent Mater.

[47] Skóra B, Krajewska U, Nowak A, Dziedzic A, Barylyak A, Kus-Liśkiewicz M (2021). Noncytotoxic silver nanoparticles as a new antimicrobial strategy. Sci Rep.

[48] Paladini F, Pollini M (2019). Antimicrobial silver nanoparticles for wound healing application: progress and future trends. Materials.

[49] Maeda H (2021). The 35th Anniversary of the Discovery of EPR Effect: a new wave of nanomedicines for tumor-targeted drug delivery—personal remarks and future prospects. J Pers Med.

[50] Singh AV, Bansod G, Mahajan M, Dietrich P, Singh SP, Rav K, Thissen A, Bharde AM, Rothenstein D, Kulkarni S, Bill J (2023). Digital transformation in toxicology: improving communication and efficiency in risk assessment. ACS Omega.

[51] https://www.who.int/news-room/fact-sheets/detail/cancer. Retrieved on 10 December 2023.

[52] Srivastava P, Kumar A (2022). Nano-cryospray: an adjuvant assisted approach to increase the efficacy of cryospray. Cryobiology.

[53] Yu Z, Gao L, Chen K, Zhang W, Zhang Q, Li Q, Hu K (2021). Nanoparticles: a new approach to upgrade cancer diagnosis and treatment. Nanoscale Res Lett.

[54] Severino P, De Hollanda LM, Santini A, Reis LV, Souto SB, Souto EB, Silva AM. Advances in nanobiomaterials for oncology nanomedicine. In: Nanobiomaterials in cancer therapy. William Andrew Publishing; 2016. pp. 91–115. https://www.sciencedirect.com/science/article/abs/pii/B9780323428637000049.

[55] Das CA, Kumar VG, Dhas TS, Karthick V, Kumar CV (2023). Nanomaterials in anticancer applications and their mechanism of action—a review. Nanomed Nanotechnol Biol Med.

[56] Cheng Z, Li M, Dey R, Chen Y (2021). Nanomaterials for cancer therapy: current progress and perspectives. J Hematol Oncol.

[57] Sahai N, Gogoi M, Ahmad N (2021). Mathematical modeling and simulations for developing nanoparticle-based cancer drug delivery systems: a review. Curr Pathobiol Rep.

[58] Li Y, Zheng X, Chu Q (2021). Bio-based nanomaterials for cancer therapy. Nano Today.

[59] Ahlawat J, Guillama Barroso G, Masoudi Asil S, Alvarado M, Armendariz I, Bernal J, Carabaza X, Chavez S, Cruz P, Escalante V, Estorga S, Narayan M (2020). Nanocarriers as potential drug delivery candidates for overcoming the blood–brain barrier: challenges and possibilities. ACS Omega.

[60] Zhu R, Zhang F, Peng Y, Xie T, Wang Y, Lan Y (2022). Current progress in cancer treatment using nanomaterials. Front Oncol.

[61] Wu J (2021). The enhanced permeability and retention (EPR) effect: the significance of the concept and methods to enhance its application. J Pers Med.

[62] Zhao T, Wu W, Sui L, Huang Q, Nan Y, Liu J (2022). Reactive oxygen species-based nanomaterials for the treatment of myocardial ischemia reperfusion injuries. Bioact Mater.

[63] Yew YP, Shameli K, Miyake M, Khairudin NBBA, Mohamad SEB, Naiki T, Lee KX (2020). Green biosynthesis of superparamagnetic magnetite Fe_3_O_4_ nanoparticles and biomedical applications in targeted anticancer drug delivery system: a review. Arab J Chem.

[64] Singh AV, Rosenkranz D, Ansari MHD, Singh R, Kanase A, Singh SP, Johnston B, Tentschert J, Laux P, Luch A (2020). Artificial intelligence and machine learning empower advanced biomedical material design to toxicity prediction. Adv Intell Syst.

[65] Haratani K, Hayashi H, Chiba Y, Kudo K, Yonesaka K, Kato R, Kaneda H, Hasegawa Y, Tanaka K, Takeda M, Nakagawa K (2018). Association of immune-related adverse events with nivolumab efficacy in non–small-cell lung cancer. JAMA Oncol.

[66] Sharma P, Allison JP (2015). Immune checkpoint targeting in cancer therapy: toward combination strategies with curative potential. Cell.

[67] Nam J, Son S, Park KS, Zou W, Shea LD, Moon JJ (2019). Cancer nanomedicine for combination cancer immunotherapy. Nat Rev Mater.

[68] Irvine DJ, Dane EL (2020). Enhancing cancer immunotherapy with nanomedicine. Nat Rev Immunol.

[69] Xu E, Saltzman WM, Piotrowski-Daspit AS (2021). Escaping the endosome: assessing cellular trafficking mechanisms of non-viral vehicles. J Control Release.

[70] Yao Y, Zhou Y, Liu L, Xu Y, Chen Q, Wang Y, Wu S, Deng Y, Zhang J, Shao A (2020). Nanoparticle-based drug delivery in cancer therapy and its role in overcoming drug resistance. Front Mol Biosci.

[71] Peer D, Karp JM, Hong S, Farokhzad OC, Margalit R, Langer R. Nanocarriers as an emerging platform for cancer therapy. Nano Enabled Med Appl. 2020;61–91. https://www.taylorfrancis.com/chapters/edit/10.1201/9780429399039-2/nanocarriers-emerging-platform-cancertherapy-dan-peer-jeffrey-karp-seungpyo-hong-omid-farokhzad-rimona-margalit-robert-langer. 10.1038/nnano.2007.387 18654426

[72] Allen S, Osorio O, Liu YG, Scott E (2017). Facile assembly and loading of theranostic polymersomes via multi-impingement flash nanoprecipitation. J Control Release.

[73] Yi S, Zhang X, Sangji MH, Liu Y, Allen SD, Xiao B, Bobbala S, Braverman CL, Cai L, Hecker PI, DeBerge M, Scott EA (2019). Surface engineered polymersomes for enhanced modulation of dendritic cells during cardiovascular immunotherapy. Adv Funct Mater.

[74] Allen S, Vincent M, Scott E (2018). Rapid, scalable assembly and loading of bioactive proteins and immunostimulants into diverse synthetic nanocarriers via flash nanoprecipitation. JoVE (J Vis Exp).

[75] Markwalter CE, Pagels RF, Wilson BK, Ristroph KD, Prud'homme RK (2019). Flash nanoprecipitation for the encapsulation of hydrophobic and hydrophilic compounds in polymeric nanoparticles. JoVE (J Vis Exp).

[76] Carugo D, Bottaro E, Owen J, Stride E, Nastruzzi C (2016). Liposome production by microfluidics: potential and limiting factors. Sci Rep.

[77] Dong Z, Kang Y, Yuan Q, Luo M, Gu Z (2018). H_2_O_2_-responsive nanoparticle based on the supramolecular self-assemble of cyclodextrin. Front Pharmacol.

[78] Xiong Q, Cui M, Yu G, Wang J, Song T (2018). Facile fabrication of reduction-responsive supramolecular nanoassemblies for co-delivery of doxorubicin and sorafenib toward hepatoma cells. Front Pharmacol.

[79] Gharnas-Ghamesh H (2021). Anticancer activity of doxorubicin loaded PBMA-b-POEGMA micelles against MCF7 breast cancer cells and HepG2 liver cancer cells. Jorjani Biomed J.

[80] Behl A, Solanki S, Paswan SK, Datta TK, Saini AK, Saini RV, Parmar VS, Thakur VK, Malhotra S, Chhillar AK (2023). Biodegradable PEG-PCL nanoparticles for co-delivery of MUC1 inhibitor and doxorubicin for the confinement of triple-negative breast cancer. J Polym Environ.

[81] Ahmad Shariff SH, Wan Abdul Khodir WK, Abd Hamid S, Haris MS, Ismail MW (2022). Poly (caprolactone)-b-poly (ethylene glycol)-based polymeric micelles as drug carriers for efficient breast cancer therapy: a systematic review. Polymers.

[82] Xiang Z, Guan X, Ma Z, Shi Q, Panteleev M, Ataullakhanov FI (2022). Bioactive engineered scaffolds based on PCL-PEG-PCL and tumor cell-derived exosomes to minimize the foreign body reaction. Biomater Biosyst.

[83] Niu K, Yao Y, Xiu M, Guo C, Ge Y, Wang J (2018). Controlled drug delivery by polylactide stereocomplex micelle for cervical cancer chemotherapy. Front Pharmacol.

[84] Chen X, Zhao L, Kang Y, He Z, Xiong F, Ling X, Wu J (2018). Significant suppression of non-small-cell lung cancer by hydrophobic poly (ester amide) nanoparticles with high docetaxel loading. Front Pharmacol.

[85] Wu J, Yuan J, Ye B, Wu Y, Xu Z, Chen J, Chen J (2018). Dual-responsive core crosslinking glycopolymer-drug conjugates nanoparticles for precise hepatocarcinoma therapy. Front Pharmacol.

[86] Xu J, Zhang Y, Xu J, Wang M, Liu G, Wang J, Zhao X, Qi Y, Shi J, Cheng K, Li Y, Nie G (2019). Reversing tumor stemness via orally targeted nanoparticles achieves efficient colon cancer treatment. Biomaterials.

[87] Panowski S, Bhakta S, Raab H, Polakis P, Junutula JR. Site-specific antibody drug conjugates for cancer therapy. In: MAbs 2014, vol. 6, No. 1. Taylor & Francis; pp. 34–45. https://www.tandfonline.com/doi/full/10.4161/mabs.27022. 10.4161/mabs.27022 PMC3929453 24423619

[88] Paik PK, Kim RK, Ahn L, Plodkowski AJ, Ni A, Donoghue MT, Jonsson P, Villalona-Calero M, Ng K, McFarland D, Fiore JJ, Rudin CM (2020). A phase II trial of albumin-bound paclitaxel and gemcitabine in patients with newly diagnosed stage IV squamous cell lung cancers. Clin Cancer Res.

[89] Parmar K, Patel J, Pathak Y. Factors affecting the clearance and biodistribution of polymeric nanoparticles. In: Pharmacokinetics and pharmacodynamics of nanoparticulate drug selivery systems. Cham: Springer International Publishing; 2022. pp. 261–272). https://link.springer.com/chapter/10.1007/978-3-030-83395-4_14.

[90] Zhang P, Meng J, Li Y, Yang C, Hou Y, Tang W, McHugh KJ, Jing L (2021). Nanotechnology-enhanced immunotherapy for metastatic cancer. Innov.

[91] Goldberg MS (2019). Improving cancer immunotherapy through nanotechnology. Nat Rev Cancer.

[92] Khalil DN, Smith EL, Brentjens RJ, Wolchok JD (2016). The future of cancer treatment: immunomodulation, CARs and combination immunotherapy. Nat Rev Clin Oncol.

[93] Hickey JW, Vicente FP, Howard GP, Mao HQ, Schneck JP (2017). Biologically inspired design of nanoparticle artificial antigen-presenting cells for immunomodulation. Nano Lett.

[94] Stephan MT, Stephan SB, Bak P, Chen J, Irvine DJ (2012). Synapse-directed delivery of immunomodulators using T-cell-conjugated nanoparticles. Biomaterials.

[95] Radovic-Moreno AF, Chernyak N, Mader CC, Nallagatla S, Kang RS, Hao L, Halo TL, Merkel TJ, Rische CH, Gryaznov SM (2015). Immunomodulatory spherical nucleic acids. Proc Natl Acad Sci.

[96] Zheng Y, Tang L, Mabardi L, Kumari S, Irvine DJ (2017). Enhancing adoptive cell therapy of cancer through targeted delivery of small-molecule immunomodulators to internalizing or noninternalizing receptors. ACS Nano.

[97] Gupta A, Mumtaz S, Li CH, Hussain I, Rotello VM (2019). Combatting antibiotic-resistant bacteria using nanomaterials. Chem Soc Rev.

[98] Ogunsona EO, Muthuraj R, Ojogbo E, Valerio O, Mekonnen TH (2020). Engineered nanomaterials for antimicrobial applications: a review. Appl Mater Today.

[99] Singh AV, Vyas V, Salve TS, Cortelli D, Dellasega D, Podestà A, Milani P, Gade WN (2012). Biofilm formation on nanostructured titanium oxide surfaces and a micro/nanofabrication-based preventive strategy using colloidal lithography. Biofabrication.

[100] Kalelkar PP, Riddick M, García AJ (2022). Biomaterial-based antimicrobial therapies for the treatment of bacterial infections. Nat Rev Mater.

[101] Sánchez-López E, Gomes D, Esteruelas G, Bonilla L, Lopez-Machado AL, Galindo R, Cano A, Espina M, Ettcheto M, Camins A, Silva AM, Souto EB (2020). Metal-based nanoparticles as antimicrobial agents: an overview. Nanomaterials.

[102] Kalashgarani MY, Babapoor A (2022). Application of nano-antibiotics in the diagnosis and treatment of infectious diseases. Adv Appl NanoBio Technol.

[103] Zhao Y, Chen L, Wang Y, Song X, Li K, Yan X, Yu L, He Z (2021). Nanomaterial-based strategies in antimicrobial applications: Progress and perspectives. Nano Res..

[104] Nas FS, Ali M, Aminu Muhammad A (2018). Application of nanomaterials as antimicrobial agents: a review. Arch Nano Op Acc J.

[105] Gupta N, Rai DB, Jangid AK, Kulhari H, Gupta N (2019). Use of nanotechnology in antimicrobial therapy. Methods in microbiology.

[106] Yan N, Xu J, Liu G, Ma C, Bao L, Cong Y, Wang Z, Zhao Y, Xu W, Chen C (2022). Penetrating macrophage-based nanoformulation for periodontitis treatment. ACS Nano.

[107] Ibrahim A, Moodley D, Uche C, Maboza E, Olivier A, Petrik L (2021). Antimicrobial and cytotoxic activity of electrosprayed chitosan nanoparticles against endodontic pathogens and Balb/c 3T3 fibroblast cells. Sci Rep.

[108] Somayajula D, Agarwal A, Sharma AK, Pall AE, Datta S, Ghosh G (2019). In situ synthesis of silver nanoparticles within hydrogel-conjugated membrane for enhanced antibacterial properties. ACS Appl Bio Mater.

[109] Zhou L, Zhao X, Li M, Lu Y, Ai C, Jiang C, Liu Y, Pan Z, Shi J (2021). Antifungal activity of silver nanoparticles synthesized by iturin against Candida albicans in vitro and in vivo. Appl Microbiol Biotechnol.

[110] Dahm H. Silver nanoparticles in wound infections: present status and future prospects. In: Nanotechnology in skin, soft tissue, and bone infections. 2020. p. 151–168.

[111] Singh AV, Katz A, Maharjan RS, Gadicherla AK, Richter MH, Heyda J, Del Pino P, Laux P, Luch A (2023). Coronavirus-mimicking nanoparticles (CorNPs) in artificial saliva droplets and nanoaerosols: influence of shape and environmental factors on particokinetics/particle aerodynamics. Sci Total Environ.

[112] Dutt Y, Pandey RP, Dutt M, Gupta A, Vibhuti A, Vidic J, Raj VS, Chang CM, Priyadarshini A (2023). Therapeutic applications of nanobiotechnology. J Nanobiotechnol.

[113] Saleemi MA, Kong YL, Yong PVC, Wong EH (2022). An overview of antimicrobial properties of carbon nanotubes-based nanocomposites. Adv Pharm Bull.

[114] Shankar S, Teng X, Rhim JW (2014). Properties and characterization of agar/CuNP bionanocomposite films prepared with different copper salts and reducing agents. Carbohyd Polym.

[115] Naradala J, Allam A, Tumu VR, Rajaboina RK (2021). Antibacterial activity of copper nanoparticles synthesized by *Bambusa arundinacea* leaves extract. Biointerface Res Appl Chem.

[116] Besinis A, De Peralta T, Handy RD (2014). The antibacterial effects of silver, titanium dioxide and silica dioxide nanoparticles compared to the dental disinfectant chlorhexidine on *Streptococcus mutans* using a suite of bioassays. Nanotoxicology.

[117] Vindhya PS, Kavitha VT. Comparative study of antibacterial activity of zinc oxide and copper oxide nanoparticles synthesized by green method. In: AIP conference proceedings, vol. 2369, no. 1. AIP Publishing; 2021.

[118] Sui M, Zhang L, Sheng L, Huang S, She L (2013). Synthesis of ZnO coated multi-walled carbon nanotubes and their antibacterial activities. Sci Total Environ.

[119] Yu B, Wang Z, Almutairi L, Huang S, Kim MH (2020). Harnessing iron-oxide nanoparticles towards the improved bactericidal activity of macrophage against Staphylococcus aureus. Nanomed Nanotechnol Biol Med.

[120] Brunet L, Lyon DY, Hotze EM, Alvarez PJ, Wiesner MR (2009). Comparative photoactivity and antibacterial properties of C60 fullerenes and titanium dioxide nanoparticles. Environ Sci Technol.

[121] Rodríguez-González V, Obregón S, Patrón-Soberano OA, Terashima C, Fujishima A (2020). An approach to the photocatalytic mechanism in the TiO_2_-nanomaterials microorganism interface for the control of infectious processes. Appl Catal B.

[122] Kumar P, Saini M, Dehiya BS, Sindhu A, Kumar V, Kumar R, Lamberti L, Pruncu CI, Thakur R (2020). Comprehensive survey on nanobiomaterials for bone tissue engineering applications. Nanomaterials.

[123] Prabhu S, Poulose EK (2012). Silver nanoparticles: mechanism of antimicrobial action, synthesis, medical applications, and toxicity effects. Int Nano Lett.

[124] Saravanan S, Nethala S, Pattnaik S, Tripathi A, Moorthi A, Selvamurugan N (2011). Preparation, characterization and antimicrobial activity of a bio-composite scaffold containing chitosan/nano-hydroxyapatite/nano-silver for bone tissue engineering. Int J Biol Macromol.

[125] Ahmadi F, Sodagar-Taleghani A, Ebrahimnejad P, Moghaddam SPH, Ebrahimnejad F, Asare-Addo K, Nokhodchi A. A review on the latest developments of mesoporous silica nanoparticles as a promising platform for diagnosis and treatment of cancer. Int J Pharm. 2022;122099. 10.1016/j.ijpharm.2022.122099 35961417

[126] Chen L, Zhou X, He C (2019). Mesoporous silica nanoparticles for tissue-engineering applications. Wiley Interdiscip Rev Nanomed Nanobiotechnol.

[127] Yang Y, Zhang M, Song H, Yu C (2020). Silica-based nanoparticles for biomedical applications: from nanocarriers to biomodulators. Acc Chem Res.

[128] Fathi-Achachelouei M, Knopf-Marques H, Ribeiro da Silva CE, Barthès J, Bat E, Tezcaner A, Vrana NE (2019). Use of nanoparticles in tissue engineering and regenerative medicine. Front Bioeng Biotechnol.

[129] Rizwan M, Shoukat A, Ayub A, Razzaq B, Tahir MB. Types and classification of nanomaterials. In: Nanomaterials: synthesis, characterization, hazards and safety. Elsevier; 2021. pp. 31–54. https://sciencedirect.com/science/article/abs/pii/B978012823823300001X.

[130] Rezaei B, Ghani M, Shoushtari AM, Rabiee M (2016). Electrochemical biosensors based on nanofibres for cardiac biomarker detection: a comprehensive review. Biosens Bioelectron.

[131] Barhoum A, Pal K, Rahier H, Uludag H, Kim IS, Bechelany M (2019). Nanofibers as new-generation materials: from spinning and nano-spinning fabrication techniques to emerging applications. Appl Mater Today.

[132] Wang R, Wang Z, Guo Y, Li H, Chen Z (2019). Design of a RADA16-based self-assembling peptide nanofiber scaffold for biomedical applications. J Biomater Sci Polym Ed.

[133] Cui H, Smith AL (2022). Impact of engineered nanoparticles on the fate of antibiotic resistance genes in wastewater and receiving environments: a comprehensive review. Environ Res.

